# The New Obstetric Instrument

**Published:** 1891-11

**Authors:** P. McCahey

**Affiliations:** 1413 South 10th street; Philadelphia


### THE NEW OBSTETRIC INSTRUMENT.

BY P. MCCAHEY, M. D., OF PHILADELPHIA.

The Atmospheric Tractor and the Uterine Safety Tube are designed to facilitate labor and make it safe and comparatively painless.

They are based upon the hitherto unrecognized fact that air heated and subjected to pressure is the principal factor in the completion of labor, and also in the production of many of the accidents of labor. This statement will no doubt be regarded with doubt by many thoughtful practitioners, but when they recollect that practically all the organs of the body utilize air as a mechanical agent in the performance of their functions, they will see no reason for assuming that the uterus should be denied the assistance of the same potent force.

Prior to the rupture of the membranes there is probably no free air within the uterine cavity, although there is doubtless a considerable quantity of it contained in solution in the liquor annii. Boyles' experiments in the 17th century demonstrated that ova of silk worms would not grow when the air was prevented of access to them, and there is no reason to suppose that the human ovum can exist without air.

After the rupture of the membranes, however, free air immediately replaces the liquor annii, and as stated by me in the New York Medical Journal of June 27th, can readily be detected by percussion. Percussion of the uterine globe before the escape of the amniotic fluid giving general dullness, but percussion afterwards, yielding distinct resonance of the fundus and in spots along the sides—i. e., above and on each side of the child.

The inrta-uterine air has several important functions to perform. It helps to preserve the rounded form of the uterus and this prevents the child from being pressed upon by the muscles during the intervals between the pains. It furnishes a medium to which muscular force can be transmitted to aid in expelling the child, and it enables artificial assistance to be applied when necessary to complete delivery.

The simplest proposition in natural philosophy and from which there can be no dissent is, that if two solid or semi-solid bodies are in contact, with no air between them, they cannot be separated except by a force applied to each of 15 lbs. to the square inch of the opposing surfaces. If there was no air between the uterus and the fœtus delivery would be impossible except by a force of six hundred to eight hundred pounds.

The fact that air is between them is evident not only by percussion nor by the ease with which labor is completed, but because it can be felt coming out through the uterine safety tube during strong pains, and can be heard entering the vagina with a "squashing" sound when the fingers are quickly withdrawn after making an examination. The well-authenticated cases of children heard crying, and felt sucking within the uterus would not be possible if air were not there. Less than two months ago, in a case of face presentation, the child sucked my finger vigorously on two different occasions four hours before it was born, and while it was still entirely within the uterus. The still more remarkable cases of posthumous birth in which children were expelled hours or days after the mothers were dead and after all muscular contraction had ceased, point conclusively to some other force than muscular energy as the expulsive agent.

As the greater portion of the uterus at times projects beyond the border of the diaphragm it is removed to a great extent from the direct action of that muscle. Then the abdominal muscles have been dilated continuously for nine months and are correspondingly weakened.

It is evident therefore that some other agent must furnish part of the expulsive mechanism.

That agent is the intra-uterine air, which as I have shown replaces the liquor annii. Its mode of action is briefly as follows: It becomes locked in by the descending head, and increasing in temperature expands in all directions. When the diaphragm descends it becomes compressed between the intestines in the rear and the abdominal wall in front, and then expanding in the line of least resistance, forces the child out through the vagina. If the head however, be large or unparted, or the perineum rigid, and the compression great, the consequent expansion will either rupture the perineum or rupture the uterus. No other hypothesis will account for the suddenness with which the perineum and rectum are sometimes torn through. The classical description of rupture of the uterus "a loud, tearing sound, the flattening of the abdominal tumor, the ready perception of the fœtal parts through the abdominal wall" points direct to the escape through the uterus of a volatile substance under great tension and which had previously kept the uterus in a globular form.

Other dangers are that when the intra-uterine pressure is intense some of the residual liquor annii will be forced into the child's lungs, thus asphyxiating it, or the blood is driven from the surface into the internal organs, producing death from meningetal or pulmonary hemorrhage.

The Uterine Safety Tube affords a means of obviating all these evils. With it the residual liquor annii can be drawn off, thus removing all possibility of occlusion of the air passages with fluid, and with it the internal pressure can be lowered whenever danger threatens either the mother or the child. It is a true, automatic safety tube lessening the pain consequent upon parturition and enabling the physician to deliver alive and vigorous, every child that is alive when the patient comes under his care.

The Atmospheric Tractor is a rubber disc which when applied to the presenting part by atmospheric pressure furnishes a means of traction without injury to either the mother or the child. It is affixed to the head by the pressure which that head must meet anyhow during its entrance into the world. It cannot exert any injurious pressure. It is uniformly applied over five square inches of the surface, and the force exerted by it is evenly divided over every particle of that surface. It does not compress the head, does not mark the surface, does not hurt the scalp, does not injure the mother. It does furnish a safe and scientific means of assisting parturition with benefit to both mother and child.

The Cranial Sound Transmitter is not an obstetric instrument, but it will be of value and interest to every obstetrician and general practitioner. It will enable the cranial, voice, air, and heart sounds to be recognized in health, and their modifications noted in disease. With it cranial auscultation and auscultatory percussion will become as general and valuable in the diagnosis and treatment of intra-cranial diseases as similar procedures now are in the management of thoracic affections.

1413 South 10th street.

